# PLK1 and β-TrCP-Dependent Ubiquitination and Degradation of Rap1GAP Controls Cell Proliferation

**DOI:** 10.1371/journal.pone.0110296

**Published:** 2014-10-17

**Authors:** Dejie Wang, Pingzhao Zhang, Kun Gao, Yan Tang, Xiaofeng Jin, Yuanyuan Zhang, Qing Yi, Chenji Wang, Long Yu

**Affiliations:** 1 State Key Laboratory of Genetic Engineering, School of Life Sciences, Fudan University, Shanghai, P.R. China; 2 Institutes of Biomedical Sciences, Fudan University, Shanghai, P.R. China; 3 Department of Gastroenterology, Jiangxi Institute of Gastroenterology & Hepatology, the First Affiliated Hospital of Nanchang University, Nanchang, Jiangxi Province, P. R. China; 4 Department of Lymphoma and Myeloma, Division of Cancer Medicine, Center for Cancer Immunology Research, The University of Texas MD Anderson Cancer Center, Houston, Texas, United States of America; University of Minnesota, United States of America

## Abstract

Rap1GAP is a GTPase-activating protein (GAP) that specifically stimulates the GTP hydrolysis of Rap1 GTPase. Although Rap1GAP is recognized as a tumor suppressor gene and downregulated in various cancers, little is known regarding the regulation of Rap1GAP ubiquitination and degradation under physiological conditions. Here, we demonstrated that Rap1GAP is ubiquitinated and degraded through proteasome pathway in mitosis. Proteolysis of Rap1GAP requires the PLK1 kinase and β-TrCP ubiquitin ligase complex. We revealed that PLK1 interacts with Rap1GAP *in vivo* through recognition of an SSP motif within Rap1GAP. PLK1 phosphorylates Ser525 in conserved _524_DSGHVS_529_ degron of Rap1GAP and promotes its interaction with β-TrCP. We also showed that Rap1GAP was a cell cycle regulator and that tight regulation of the Rap1GAP degradation in mitosis is required for cell proliferation.

## Introduction

Rap1GAP is a member of a family of GTPase-activating proteins (GAPs) that specifically stimulate the GTP hydrolysis of Rap1 GTPases [1]. Rap1 is one of the Ras-like small GTPases that are critical players in signaling pathways that control cell growth, migration, and differentiation [1]. Rap1 shuttles between an inactive GDP- and active GTP-bound form. Activation of Rap1 (Rap1-GTP) is mediated by guanine nucleotide exchange factors (GEFs), including C3G, PDZ-GEF, Epac, and CalDAG. Inactivation of Rap1 is mediated by GTPase activating proteins (GAPs), including Rap1GAP and Rap1GAP2, SPA-1/SIPA1 and SIPA1L1/SPAR [2].

Rap1GAP is a tumor suppressor gene and downregulated in various cancers such as squamous cell carcinoma, renal cell carcinoma, melanoma, pancreatic cancer, and thyroid cancer [3]–[7]. Restoring Rap1GAP expression to these cancer cells inhibited cell proliferation, migration, and invasion, effects that were correlated with the inhibition of Rap1 activity. Rap1GAP expression and activity has been reported to be regulated at transcriptional and post-translational level. Down-regulation of Rap1GAP was frequently achieved by promoter hypermethylation [5], [8], [9]. A recent study revealed a novel mechanism for sustained activation of Rap1 via downregulation of microRNA-101 (miR-101). Loss of expression of miR-101 upregulates EZH2, which promotes di- or tri-methylation at lysine 27 of histone H3, resulting in chromatin condensation as well as promoter hypermethylation, thereby silencing Rap1GAP [9]. Furthermore, Rap1GAP can be phosphorylated by various protein kinases, such as PKA, GSK-3β and CDK1, in response to different signals [10]–[12].

Protein ubiquitination has emerged as a fundamental mechanism for regulating protein half-life and activity. The specificity of the ubiquitination reaction is achieved by the E3 ubiquitin ligases (E3), which mediate the transfer of ubiquitin from E2 ubiquitin-conjugating enzymes (E2) to the substrates [13]. The ubiquitin and proteasome system is a major regulatory mechanism for diverse cellular pathways, such as endocytosis, apoptosis, DNA damage response, and cell cycle regulation. Two E3 ubiquitin ligase families are prominent in cell cycle regulation and mediate the timely and precise ubiquitin-proteasome-dependent degradation of key cell cycle regulators: the APC/C (anaphase promoting complex or cyclosome) and the SCF (Skp1/Cul1/F-box protein) complex [14]. The β-TrCP ubiquitin ligase complex is the best characterized mammalian Cullin-based ubiquitin ligases, consisting of the molecular scaffold Cul1, the adaptor Skp1, RING finger protein Rbx1 and an F-box protein, β-TrCP. β-TrCP provides the complex with its substrate targeting specificity-it directly interacts with substrates, and acts as an adaptor protein to bridge substrates to the ligase, thereby targeting them for destruction [15]. The majority of the β-TrCP substrates contain a DSGxxS/T degron, and β-TrCP recognizes this degron when both Ser/Thr are phosphorylated [15]. The β-TrCP ligase complex is a key enzyme that acts with cell cycle-related kinases (CDKs, PLK1, Chk1 and others) to control timely and precise proteolysis of cell cycle proteins and to mediate the cell cycle transitions [16]. The cell cycle regulators known to be degraded by β-TrCP ligase include Emi1, Cdc25A, Wee1, Bora, FANCM [16], and the list is still growing.

In this study, we report that during mitosis, Rap1GAP undergoes ubiquitin-dependent degradation, which is regulated by β-TrCP ubiquitin ligase and the Polo-like kinase 1 (PLK1). Importantly, Rap1GAP degradation is required for cell proliferation.

## Materials and Methods

### Cell Culture and transfection

U2OS, 293T, and HeLa cells were obtained from the American Type Culture Collection. U2OS, HeLa, and 293T cells were maintained in DMEM with 10% FBS. Cells were transiently transfected using Lipofectamine 2000 (Invitrogen, USA) according to manufacturer’s instructions.

### Expression constructs

Human Rap1GAP construct was kindly provided by Judy L. Meinkoth (University of Pennsylvania), and subcloned into pCMV-HA, pcDNA3.0-Flag, or pGEX-4T-2 vector. Flag-β-TrCP1 and β-TrCP2 were kindly provided by Michele Pagano (New York University). Myc-PLK1 WT and KD mutant were kindly provided by Dr. Erich A. Nigg (University of Basel, Switzerland), and subcloned into pGEX-4T-2 vector. Myc-β-TrCP1, Myc-Skp2, Myc-Fbw5, Myc-Cdc20, and Myc-Cdh1 were kindly provided by Dr. Yue Xiong (University of North Carolina at Chapel Hill). Rap1GAP mutants were generated using the KOD-Plus Mutagenesis Kit (Toyobo, Japan).

### RNA Interference

The siRNA oligos were purchased from Genepharma (Shanghai, China). The siRNA oligos sequences for Rap1GAP are: RNAi #1: 5′-GCAAGGAGCAUUUCAAUUAdTdT-3′; RNAi #2: 5′-GCUGAUCAAUGCUGAAUAUdTdT-3′. The siRNA oligos sequence for β-TrCP 1/2 is: 5′-GUGGAAUUUGUGGAACAUCdTdT-3′; The siRNA oligos sequence for PLK1 is: 5′-CCUUGAUGAAGAAGAUCACdTdT-3′; The siRNA oligos sequence for GSK-3β is: 5′-GCAAAUCAGAGAAAUGAACdTdT-3′; The siRNA oligos sequence for control is: 5′-UUCUCCGAACGUGUCACGUdTdT-3′.

### Antibodies

The following antibodies were used: Rap1GAP (sc-130646; Santa Cruz), Rap1GAP (sc-10331; Santa Cruz), p53 (sc-126; Santa Cruz), Aurora B (sc-25426; Santa Cruz), PLK1 (sc-17783; Santa Cruz), GSK-3β (sc-81462; Santa Cruz), Cyclin B (4135; Cell Signaling), Wee1(sc-325; Santa Cruz), p-histone H3 (sc-8656-R; santa Cruz), β-TrCP (373400; Invitrogen), Myc (9E10; Sigma), FLAG (M2; Sigma), HA (MM5–101R; Convance), Actin (AC-74; Sigma).

### Cell synchronization

For synchronization of cells in G1/S phase, HeLa cells were incubated with 2 mM thymidine for 16 hours twice with an intervening 8 hours of incubation in fresh medium without thymidine. For synchronization of cells in M phase, double thymidine-treated HeLa cells were released into medium containing 150 ng/ml nocodazole. The cell cycle synchronization was confirmed by flow cytometry after propidium iodide staining.

### Immunoprecipitation

Cells were lysed with 1X cell lysis buffer (Cell Signaling, USA), and the lysate was centrifuged. The supernatant was precleared with protein A/G beads (Sigma, USA), and incubated with indicated antibody overnight at 4°C. Thereafter protein A/G beads were applied, also at 4°C. After 2 h of incubation, pellets were washed 5 times with lysis buffer, resuspended in sample buffer, and analyzed by SDS–PAGE.

### Immunoblotting

Cell lysates or immunoprecipitates were subjected to SDS-PAGE, and proteins were transferred to nitrocellulose membranes (GE Healthcare, USA). The membrane was blocked in Tris-buffered saline (TBS, pH 7.4) containing 5% non-fat milk and 0.1% Tween-20, washed twice in TBS containing 0.1% Tween-20, and incubated with primary antibody for 2 hours and followed by secondary antibody for 1 hour at room temperature. Afterward, the proteins of interest were visualized using an enhanced chemiluminescence system (Santa Cruz, USA).

### *In Vitro* Kinase assay

Immunoprecipitated and GST-protein complexes were incubated in a kinase buffer containing 50 mM Tris-HCl pH 7.5, 10 mM manganese chloride, and 100 mM [γ-32P]ATP (500 c.p.m./pmol). After incubation for 30 min at 30°C, the reactions were terminated by addition of SDS sample buffer, and the proteins separated by SDS-PAGE and analyzed by autoradiography.

### Colony formation assay

HeLa cells were seeded at the same density in 6-well dishes (1×10^3^ cells/dish). After 20 h, cells were transiently transfected with Flag-Rap1GAP (WT or S525/529A mutant) or control vector. Transfectants were selected using G418 (750 µg/ml) for 2 to 3 weeks and stained with crystal violet. The total number of colonies in each well from three independent transfections was counted.

### Brdu incorporation assay

Proliferation was measured using a colorimetric 5-bromo-2-deoxyuridine (BrdU) Cell Proliferation ELISA Kit (Roche, Japan). Cells were incubated with BrdU labeling solution for additional 6 h at 37°C, then fixed and denatured by FixDenat solution. After incubation with anti-BrdU-peroxidase working solution, substrate solution was added until the color development was sufficient for photometric detection. H_2_SO_4_ (1mM) was applied to stop the reaction. Absorbance was measured at 450 nm using an automatic ELISA reader.

### Statistical analysis

The data are expressed as the mean ± S.D. from three independent experiments. Statistical analysis was performed using a two-sided *t*-test. A value of p<0.05 was considered statistically significant.

## Results

### Rap1GAP is degraded during mitosis

**Figure 1:**
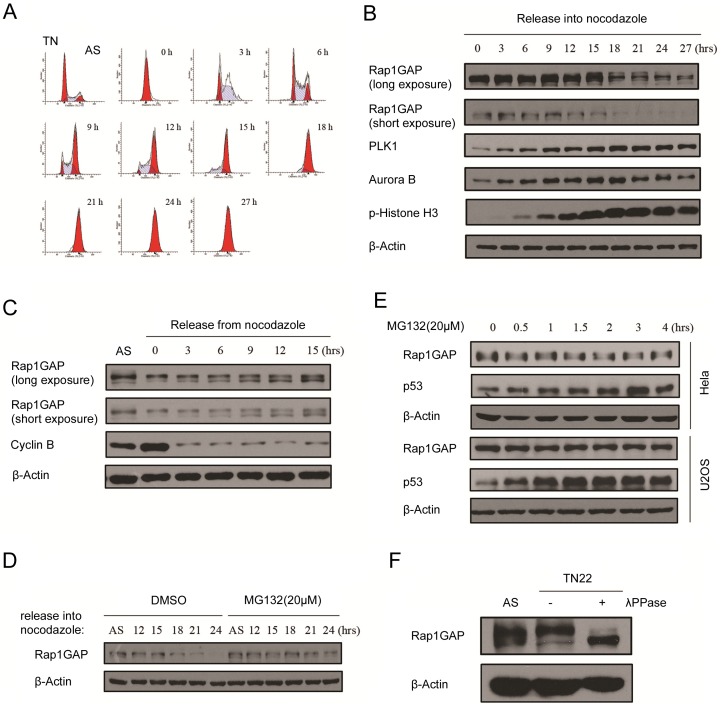
Rap1GAP is degraded during mitosis. (A and B) HeLa cells were synchronized by double thymidine block followed by release into nocodazole-containing media and harvested at the indicated times. Cell cycle profile was assayed by FACS with propidium iodine staining (A). Protein levels were analyzed by immunoblotting (B). (C) Nocodazole-arrested HeLa cells were released into fresh medium, and the Rap1GAP status was monitored at indicated times. (D) HeLa cells were arrested using nocodazole at the indicated times, and 20 µM MG132 was added during the last 4 h of the nocodazole treatment before the cells were harvested. (E) HeLa cells or U2OS cells were treated with 20 µM MG132 and harvested at the indicated times. Protein levels were examined by immunoblotting. (F) HeLa cells that were synchronized by double thymidine block followed by release into nocodazole-containing media, and harvested at 22 h. The cell lysates were incubated with or without λ-phosphatase (λ-PPase). The phosphorylation state of Rap1GAP was analyzed by immunoblotting.

We examined the Rap1GAP protein level through the cell cycle and compared it to other key cell cycle regulators. HeLa cells were arrested at the G1/S boundary by a double thymidine treatment and then released into nocodazole-containing media. The cell cycle profile of released cells was analyzed by FACS (Fig. 1A). The mitotic time-points were determined by phosphorylated histone H3 (p-histone H3) level. As shown in Fig. 1B, p-histone H3 was not detectable at the G1-S boundary but gradually accumulated as cells progressed into S and G2, peaking at 21 hours after release, a time at which the majority of cells had entered mitosis (Fig. 1B). PLK1 and Aurora B, two key mitotic kinase levels were low during interphase but high during mitosis (Fig. 1B). In contrast, the Rap1GAP protein level was consistent at the G1/S and S phase, and then gradually showed motility shifts, peaking at 15 h after release, and gradually decreased in mitosis (Fig. 1B). When cells were released from mitotic arrest, the Rap1GAP level was restored by the time the cells exited mitosis (Fig. 1C). Furthermore, a significant fraction of Rap1GAP was restored following exposure of the arrested cells to the proteasome inhibitor MG132, suggesting that the proteasome pathway is involved in Rap1GAP degradation in mitosis (Fig. 1D). To determine whether Rap1GAP was specifically degraded in mitosis, we treated the unsynchronized HeLa cells (91% in interphase) with MG132. p53, a short-lived tumor suppressor protein, was rapidly stabilized with MG132 treatment. In contrast, the Rap1GAP protein level remained constant (Fig. 1E). Similar results were obtained in U2OS cells (Fig. 1E). Finally, the Rap1GAP mobility shifts were observed throughout the cell cycle, and these mobility shifts were abolished with λ-phosphatase treatment (Figure 1F). This loss suggests that the post-translational modification of Rap1GAP resulted from phosphorylation. Taken together, these data demonstrated that Rap1GAP was phosphorylated and degraded by the proteasome pathway during mitosis.

### The β-TrCP component of the SCF E3 ligase mediates degradation of RAP1GAP

**Figure 2:**
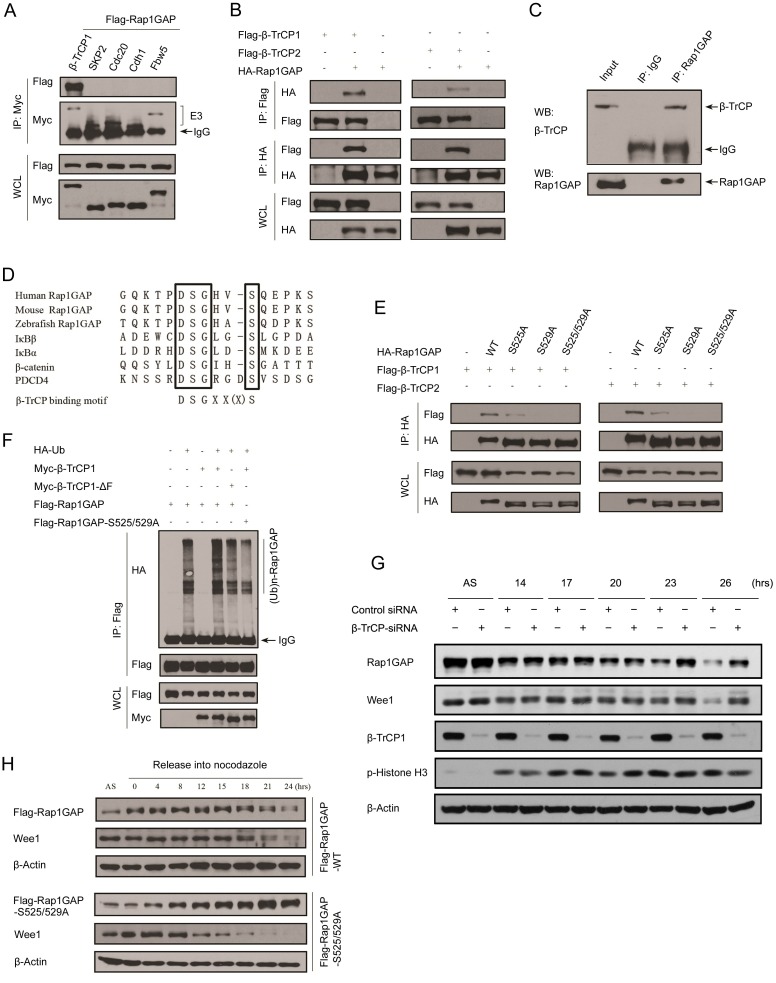
Rap1GAP is ubiquitinated and degraded in a β-TrCP dependent manner. (A) 293T cells were co-transfected with Flag-Rap1GAP and the indicated Myc-tagged E3 ligases constructs (β-TrCP1, SKP2, Cdc20, Cdh1 and Fbw5). At 24 h after transfection, cells were harvested and lysed. Cell lysates were subjected to immunoprecipitation with anti-Myc antibody. The immunoprecipitates were immunoblotted with anti-Flag and anti-Myc antibodies, respectively. (B) 293T cells were co-transfected with HA-Rap1GAP and Flag- β-TrCP1/2 constructs. Cell lysates were prepared and subjected to immunoprecipitation with anti-HA or anti-Flag antibodies, respectively. (C) Endogenous Rap1GAP interacts with endogenous β-TrCP in HeLa cells. At the 4 h before harvesting, cells were treated with the proteasome inhibitor, MG132. HeLa cell lysates were incubated with protein A/G sepharose conjugated with either control IgG or Rap1GAP antibody. The immunoprecipitates were immunoblotted with Rap1GAP or β-TrCP antibodies, respectively. (D) Alignment of amino acids corresponding to the DSGxxS sequence with Rap1GAP1 orthologs and other β-TrCP substrates. (E) 293T cells were co-transfected with Flag-β-TrCP1/2 and wild type HA-Rap1GAP or SA mutant (S525A, S529A and S525/529A) constructs. At 24 h after transfection, cells were harvested and lysed. Cell lysates were subjected to immunoprecipitation with anti-HA antibody. The immunoprecipitates were immunoblotted with anti-Flag and anti-HA antibodies, respectively. (F) Flag-Rap1GAP (WT or S525/529A), HA-ubiquitin, and β-TrCP1 (WT or ΔF Box mutant) constructs were co-transfected into 293T cells. Rap1GAP proteins were immunoprecipitated by anti-Flag antibody. The polyubiquitinated forms of Rap1GAP were immunoblotted with anti-HA antibody. (G) Control siRNA or β-TrCP-specific siRNA were transfected in HeLa cells that were synchronized by double thymidine block followed by release into nocodazole-containing media for the indicated times. Cell lysates were subjected immunoblotting with the indicated antibodies. (H) Flag-Rap1GAP wild type or S525/529A mutant was transfected into HeLa cells that were synchronized by double thymidine block followed by release into nocodazole-containing media for the indicated times. Cell lysates were subjected to immunoblotting with the indicated antibodies.

The SCF complex plays a prominent role at the transition between the stages of the cell cycle via its various regulatory subunits, such as Skp2, Fbw5, and β-TrCP. APC/C, regulated by the Cdc20 or Cdh1 subunits, also has a crucial role during mitosis. We hypothesized that one of these complexes might be responsible for the mitotic degradation of Rap1GAP. To answer this question, we first examined the ability of five E3 ligase subunits (β-TrCP1, Skp2, Fbw5, Cdh1 and Cdc20) to interact with Rap1GAP. As shown in Fig. 2A, β-TrCP1 was the only subunit that interacted with Rap1GAP. In a reciprocal co-immunoprecipitation experiment, we also demonstrated that β-TrCP2, a paralog that shares identical biochemical properties and substrates with β-TrCP1, also interacted with Rap1GAP (Fig. 2B). Next, we confirmed whether β-TrCP interacts with Rap1GAP at an endogenous level. When endogenous Rap1GAP in nocodazole-arrested HeLa cells was immunoprecipitated by anti-Rap1GAP antibody, endogenous β-TrCP was also detected in the immunoprecipitate by immunoblotting (Fig. 2C). Therefore, these results indicated β-TrCP interacted with Rap1GAP *in vivo*.

The WD40 β-propeller structure of β-TrCP binds its substrates through a diphosphorylated degradation motif (phosphodegron) with the consensus sequence DpSGXX(X)pS/T or similar variants. Inspection of the Rap1GAP amino acid sequence revealed a sequence resembling the motif of the β-TrCP binding pattern. The motif surrounding Ser525 and Ser529 is highly conserved in vertebrate orthologs of Rap1GAP (Fig. 2D). To investigate whether Rap1GAP binds β-TrCP via this motif, we generated three Rap1GAP mutants in which Ser525 or Ser529 was mutated respectively or in combination to Ala. After that, we tested their binding to β-TrCP-1/2. While the Rap1GAP (S525A) mutant immunoprecipitated less β-TrCP1/2 compared to wild-type Rap1GAP, Rap1GAP (S529A) or (S525/529A) mutants lost the ability to bind to β-TrCP1/2 (Fig. 2E), suggesting two Serine residues are required for Rap1GAP to bind β-TrCP1/2. We determined whether manipulation of β-TrCP levels would alter Rap1GAP ubiquitination. Indeed, the *in vivo* ubiquitination assay showed that overexpression of β-TrCP1, but not its enzyme-dead form (ΔF), enhanced the ubiquitination of exogenously expressed Rap1GAP. Furthermore, Rap1GAP (S525/529A) mutant ubiquitination was remarkably reduced (Fig. 2F). We next investigated whether β-TrCP is responsible for Rap1GAP degradation in mitosis. As shown in Fig. 2G, knockdown of β-TrCP using a siRNA abrogated the mitotic degradation of both Rap1GAP and the known β-TrCP substrate Wee1. To investigate whether the Rap1GAP phosphodegron is critical for mitotic degradation *in vivo*, we expressed either the wild-type or mutant Rap1GAP in HeLa cells. We found that the mutant protein did not undergo degradation in mitosis, whereas wild-type Rap1GAP protein was destabilized (Fig. 2H). Therefore, these results indicate Rap1GAP interacts with β-TrCP *in vivo,* and the phosphodegron sequence of Rap1GAP determines the β-TrCP binding and subsequent ubiquitination and degradation of Rap1GAP in mitosis.

### PLK1 interacts with Rap1GAP and phosphorylates it at Ser525

**Figure 3:**
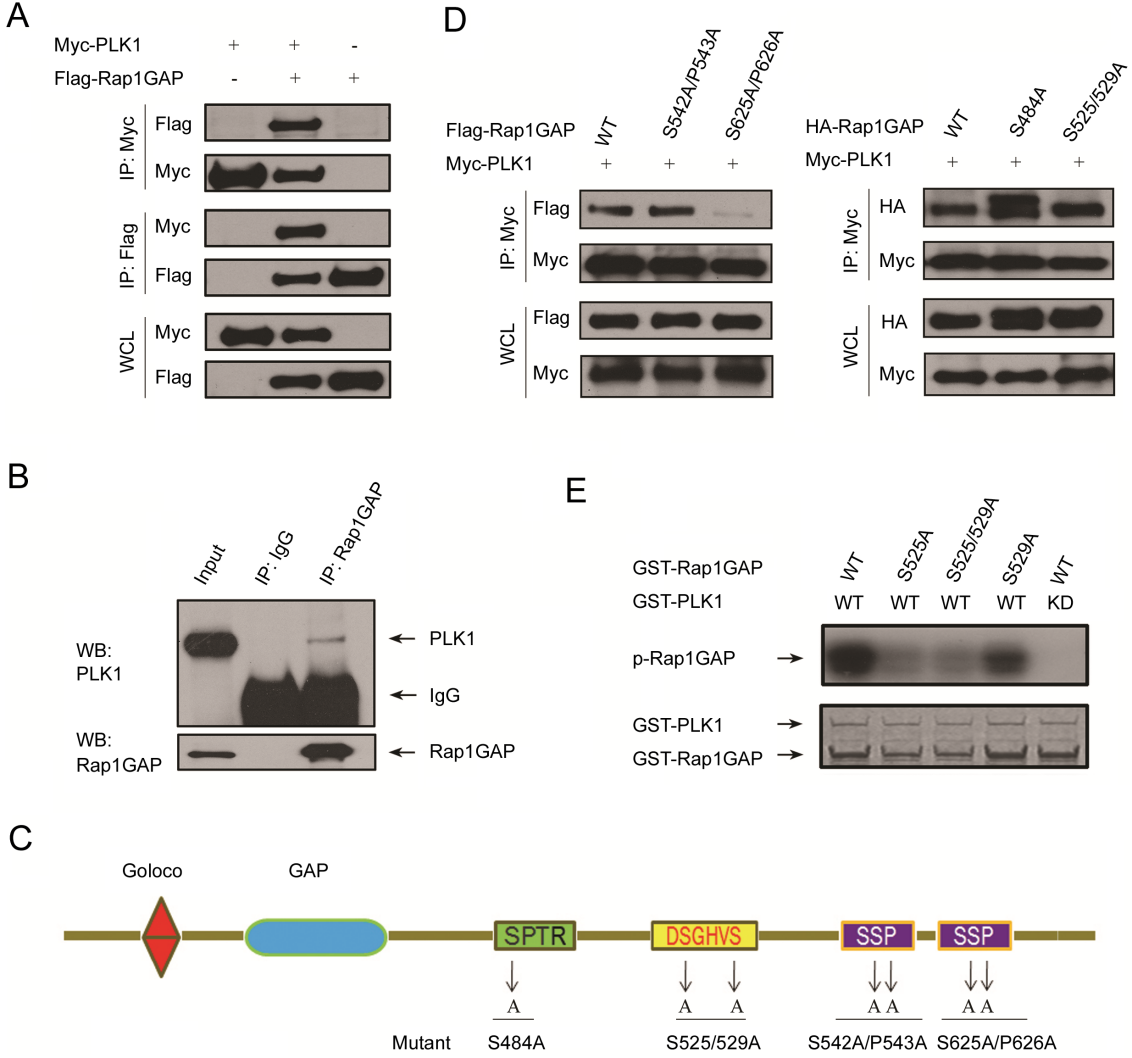
PLK1 interacts with Rap1GAP and phosphorylates it at Ser525. (A) 293T cells were co-transfected with Flag-Rap1GAP and Myc-PLK1 constructs. Cell lysates were prepared and subjected to immunoprecipitation with Flag or Myc antibodies, respectively. The immunoprecipitates were immunoblotted with anti-Flag and anti-Myc antibodies, respectively. (B) Endogenous Rap1GAP interacts with endogenous PLK1 in HeLa Cells. HeLa cells were synchronized by double thymidine block followed by release into nocodazole-containing media for 10 h. HeLa cells lysates were incubated with protein A/G sepharose conjugated with either control IgG or Rap1GAP antibody. The immunoprecipitates were immunoblotted with Rap1GAP or PLK1 antibodies, respectively. (C) Rap1GAP contains two PLK1 Polo box domain recognition motifs (S-S-P) located in the C-terminal sequence. (D) 293T cells were co-transfected with Myc-PLK1 and Flag-Rap1GAP (WT, S542A/P543A or S625A/P626A) (*left panel)*, or HA-Rap1GAP (WT, S484A or S525/529A) constructs (*right panel*). Cell lysates were prepared and subjected to immunoprecipitation with Myc antibody. The immunoprecipitates were immunoblotted with anti-Flag and anti-HA antibodies, respectively. (E) Recombinant GST-Rap1GAP wild type or SA mutants (S525A, S529A and S525/529A) were phosphorylated by purified recombinant PLK1 wild type or kinase dead mutant (KD). Visualization by autoradiography (*top panel*) revealed Rap1GAP phosphorylation by PLK1. Coomasie brilliant blue staining (*bottom panel*) protein bands for Rap1GAP and PLK1.

SCF complex ligases bind and ubiquitinate proteins that are phosphorylated at specific phosphodegron sequences. Targeting of proteins for destruction by phosphorylation provides a mechanism for linking cell cycle regulation to internal and external signaling pathways via regulating protein kinase activities [16]. Because the mitotic phosphorylation of Rap1GAP precedes its degradation, we reasoned that mitotic kinase(s) might also destabilize Rap1GAP. PLK1 is a critical kinase regulating mitotic progression and cell cycle checkpoints. Importantly, it has been shown previously to regulate several β-TrCP targets, such as Emi1, Wee1, and Claspin during mitosis [16]. Therefore, we hypothesized that PLK1 might regulate Rap1GAP degradation. To test this hypothesis, we first examined whether PLK1 can interact with Rap1GAP in cells. We co-expressed Flag-Rap1GAP and Myc-PLK1 in 293T cells and immunoprecipitated PLK1 using anti-Myc antibody. As shown in Fig. 3A, Rap1GAP was co-immunoprecipitated by PLK1. Similar results were obtained in a reciprocal co-immunoprecipitation experiment using anti-Flag antibody (Fig. 3A). Next, we wanted to confirm whether PLK1 interacts with Rap1GAP at an endogenous level. When endogenous Rap1GAP in nocodazole-arrested HeLa cells was immunoprecipitated by anti-Rap1GAP antibody, endogenous PLK1 was detected in the immunoprecipitate (Fig. 3B). Previous studies suggested that the interactions between PLK1 with its substrates are largely mediated by PLK1 Polo box domain (PBD) recognition of Ser–pSer/pThr–Pro (S–pS/pT–P) motifs within the substrates [17]. Inspection of the amino acid sequence of Rap1GAP revealed that human Rap1GAP contains two such S-S/T-P motifs located in the C-terminal sequence (Fig. 3C). To determine which motif represents the PLK1 docking site on Rap1GAP, we generated two Rap1GAP mutants, in which Ser542/Pro543 or Ser625/Pro626 was mutated to Ala (S542A/P543A and S625A/P626A, respectively) and tested their binding to PLK1. While the Rap1GAP (S542A/P543A) mutant immunoprecipitate PLK1 similar to wild-type Rap1GAP, the Rap1GAP (S625A/P626A) mutant lost the ability to bind to PLK1 (Fig. 3D, left panel), This observation suggests the second S-S/T-P motif is responsible for PLK1 binding.

It has been reported that Rap1GAP is a substrate of the CDK1 kinase in mitosis [18]. Rap1GAP Ser484 is the site phosphorylated by CDK1 *in vitro*. However, the phosphorylation status of this site does not affect the stimulation of the GTPase activity of Rap1 by Rap1GAP, and its function remains unknown [18]. Previous studies have revealed that CDK1 is the “priming” kinase that initially phosphorylates several PLK1 substrates, such as BubR1, CEP55, and Cdc25C, and generates the docking sites for the PBD. We investigated whether the CDK1 phosphorylation site in Rap1GAP was involved in PLK1 binding. Rap1GAP mutant (Ser484A) was generated and tested its binding to PLK1. However, we found that the S484A mutant immunoprecipitated PLK1 at a level similar to that immunoprecipitated by wild-type Rap1GAP (Fig. 3D, right panel), suggesting that CDK1 phosphorylation is not required for Rap1GAP binding to PLK1. Furthermore, another Rap1GAP mutant (S525/529A) that lost the ability to bind to β-TrCP, immunoprecipitated PLK1 at a level similar to that immunoprecipitated by wild-type Rap1GAP (Fig. 3D, right panel), suggesting that Rap1GAP interacted with β-TrCP and PLK1 through different sequence motifs. Finally, an *in vitro* phosphorylation assay showed that PLK1 directly phosphorylated the DSGHVS degron, as the Rap1GAP (S525/5299A) mutant lost the ability to be phosphorylated by recombinant PLK1. While the Rap1GAP (S525A) mutant was not phosphorylated by PLK1, Rap1GAP (S529A) mutant retained a level of phosphorylation similar to that of wild-type Rap1GAP (Fig. 3E). Taken together, these data suggest that Ser525 was the major site phosphorylated by PLK1.

### PLK1 is required for Rap1GAP degradation during mitosis

**Figure 4:**
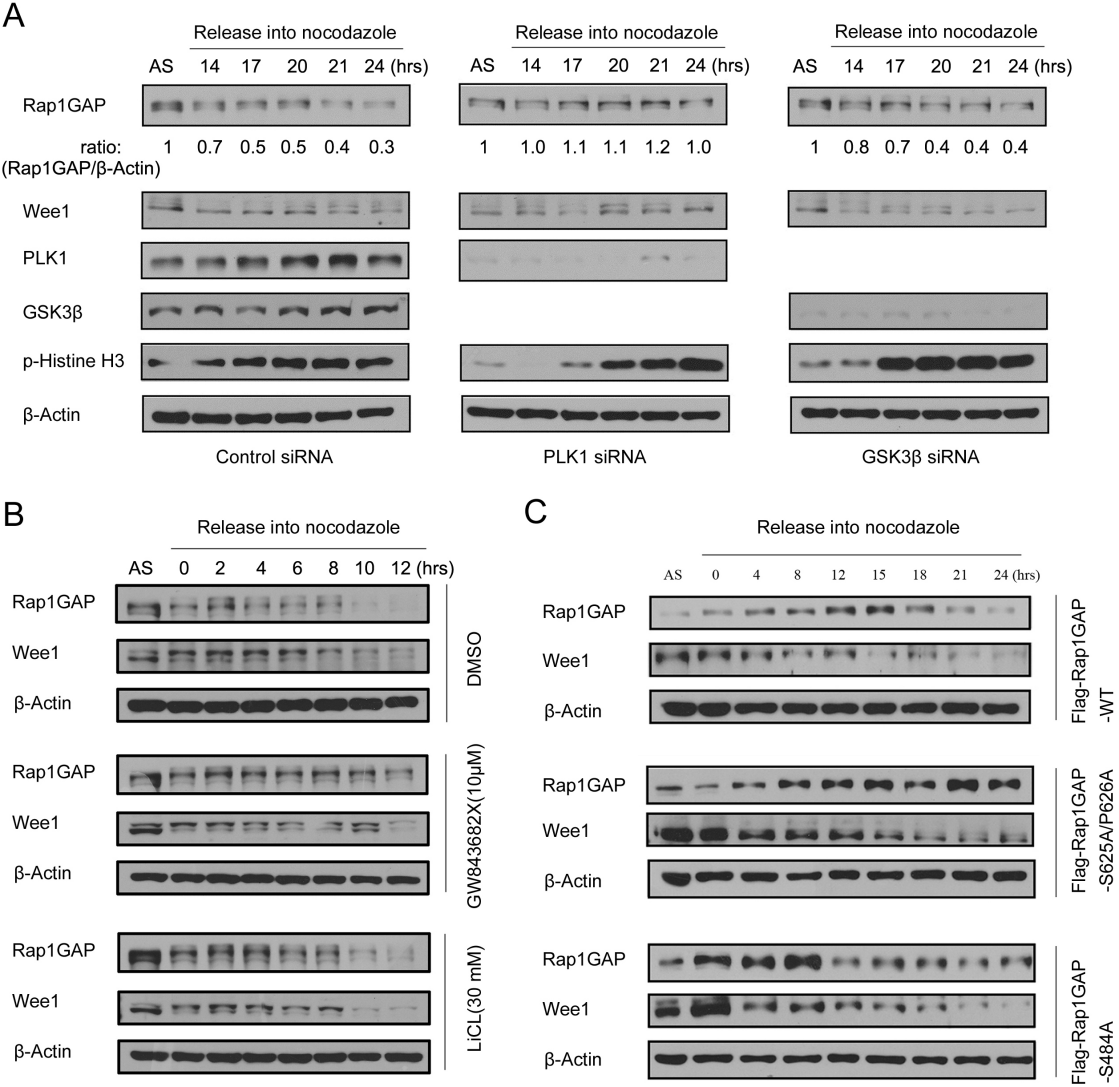
PLK1 is essential for Rap1GAP degradation in mitosis. (A) Control siRNA or PLK1-specific siRNA was transfected in Hela cells that were synchronized by double thymidine block followed by release into nocodazole-containing media for the indicated times. Cell lysates were subjected immunoblotting with the indicated antibodies. (B) HeLa cells were synchronized by double thymidine block followed by release into nocodazole-containing media for 15 h, then the cells were treated with the PLK1-specific inhibitor GW843682X (10 µM), GSK3β-specific inhibitor LiCl (10 mM), or control (DMSO) for indicated times, Cell lysates were subjected to immunoblotting with the indicated antibodies. (C) Flag-tagged Rap1GAP wild type or mutants (S484A, S625A/P626A) were transfected into HeLa cells that were synchronized by double thymidine block followed by release into nocodazole-containing media for the indicated times. Cell lysates were subjected to immunoblotting with the indicated antibodies.

Next, we investigated whether PLK1 was required for Rap1GAP degradation during mitosis. We synchronized HeLa cells by double thymidine block, knocked down PLK1 using siRNAs, and monitored the level of Rap1GAP at indicated time points. As shown in Fig. 4A, siRNA specific to PLK1 abrogated the degradation of its known substrate Wee1. The mitotic degradation of Rap1GAP was also significantly inhibited, suggesting that PLK1 is the key kinase triggering mitotic degradation of Rap1GAP. Previous studies reported that Rap1GAP protein level was dynamically regulated in thyroid-stimulating hormone (TSH)-treated thyroid cells. Upon TSH withdrawal, Rap1GAP undergoes a net increase in GSK-3β-dependent phosphorylation followed by proteasome-mediate degradation [11]. To investigate whether GSK-3β was involved in the mitotic degradation of Rap1GAP, we knocked down GSK-3β in synchronized HeLa cells like PLK1. However, GSK-3β knockdown did not affect the mitotic degradation of Rap1GAP (Fig. 4A). To rule out potentially indirect effects of siRNA treatment, we used small molecule inhibitors of PLK1 and GSK-3β. Treatment with the PLK1 inhibitor GW843682X, but not the GSK-3β inhibitor LiCl, abrogated the mitotic degradation of Rap1GAP (Fig. 4B). These results were consistent with siRNA knockdown treatment. To demonstrate whether the second SSP motif of Rap1GAP is critical for mitotic degradation, wild-type Rap1GAP or two mutants (S484A, S625A/P626A) was expressed in HeLa cells, respectively. We found that the Rap1GAP mutant (S625A/P626A) did not undergo degradation and was more stable in mitosis, whereas wild-type Rap1GAP or Rap1GAP mutant (S484A) proteins was destabilized (Fig. 4C). Together, these results further support a model in which PLK1, but not CDK1 or GSK-3β-mediated phosphorylation of RAP1GAP is a prerequisite for mitotic degradation.

### Rap1GAP degradation is required for cell proliferation

**Figure 5:**
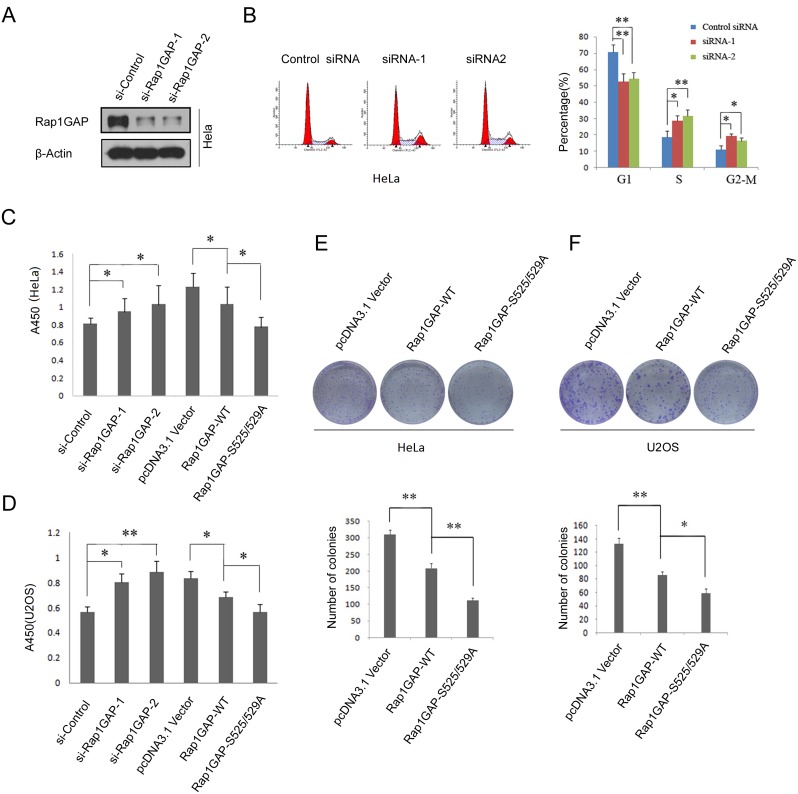
Rap1GAP degradation is required for cell proliferation. (A) HeLa cells were transiently transfected with two Rap1GAP-specific siRNAs or control siRNA. At 48 h after transfection, cell lysates were subjected to immunoblotting with Rap1GAP antibody. (B) The cell cycle profile of Rap1GAP-knocked down HeLa cells were assayed by FACS with propidium iodine staining. (C and D) The proliferative capacity of Rap1GAP-knocked down or overexpressed HeLa (C) or U2OS (D) cells was measured by BrdU ELISA assay. HeLa cells or U2OS cells were transfected with Flag-Rap1GAP WT, S525/529A mutant or control constructs, the cell proliferative capacity were measured by BrdU ELISA assay. Similar ELISA assay was performed when Rap1GAP was knocked down. (E and F) HeLa (E) or U2OS (F) cells were transfected with Flag-Rap1GAP WT, S525/529A mutant or control constructs respectively and selected with G418 for 3 weeks and outgrowth colonies were stained by crystal violet. Representative photographs of cell colonies were shown. Total numbers of the colonies from three independent experiments were counted. *P<0.05; **, p<0.001.

We analyzed the physiological function of Rap1GAP degradation. The endogenous Rap1GAP was efficiently knocked down by two specific siRNAs (Fig. 5A). The effect of Rap1GAP depletion on cell cycle distribution in HeLa cells was examined. We found that the fraction of cells in G1 was lower when Rap1GAP expression was depleted (siRNA-1 or siRNA-2) than that observed in control siRNA knocked-down cells (Fig. 5B). Similar results were seen in U20S cells (data not shown). This finding suggests that the absence of Rap1GAP may affect cell cycle transitions. Next, BrdU incorporation assay results showed that knockdown of Rap1GAP expression enhanced DNA synthesis activities in HeLa and U2OS cells (Fig. 5C and 5D). In a complementary experiment, the same amount of Flag-Rap1GAP WT or the degron mutant Rap1GAP (S525/529A) construct and a control vector were transiently overexpressed in HeLa or U2OS cells, respectively. BrdU incorporation assay showed that transfection of Rap1GAP WT or (S525/529A) mutant reduced cell proliferation when compared with cells expressing the control vector, degron mutant Rap1GAP (S525/529A) elicited a more dramatic phenotype (Fig. 5C and 5D). Finally, we investigated the potential role of Rap1GAP in cell growth using a colony formation assay. HeLa cells were transfected with Rap1GAP WT or (S525/529A) mutant and stable clones were counted. Notably, both transgenes manipulation lead to a significant reduction in colony number, but the degron-mutant Rap1GAP (S525/529A) elicited a more dramatic phenotype (Fig. 5E). Similar results were obtained in U20S cells (Fig. 5F). In summary, we concluded that Rap1GAP degradation is required for cell proliferation.

## Discussion

In the present study, Rap1GAP was identified as a cell-cycle regulator and subjected to PLK1 and β-TrCP-dependent degradation in mitosis. We found that in mitosis, PLK1 phosphorylated Rap1GAP on its conserved DSGHVS degron, which promoted its interaction with SCF-TrCP, and also facilitated its degradation. Ser525 was the major site phosphorylated by PLK1, but the mitotic kinase responsible for Ser259 phosphorylation is unknown now and await further studies. Since Rap1GAP (S529A) mutant lost the ability to bind to β-TrCP1/2, we hypothesize that other kinase(s) responsible for Ser529 phosphorylation are also for important for Rap1GAP degradation in mitosis. Our findings show that Rap1GAP can be added to the list of cell cycle regulators, such as Claspin, Wee1, Emi1, FANCM, and Bora proteins, whose ubiquitin-dependent degradation is mediated by SCF–β-TrCP and controlled by PLK1. Although we have defined the degron that is critical for SCF-β-TRCP-mediated degradation of Rap1GAP, it is possible that Rap1GAP degradation also can be regulated by other mechanisms, possibly in response to other signals and involving different determinants in the Rap1GAP protein. For example, others have reported that Rap1GAP protein levels are dynamically regulated in thyroid-stimulating hormone (TSH)-dependent thyroid cells. Upon TSH withdrawal, Rap1GAP undergoes a net increase in GSK3β-dependent phosphorylation followed by proteasome-mediated degradation [11]. However, our study showed GSK3β is not essential for Rap1GAP degradation in mitosis. Another study showed that the Gαo/i-coupled cannabinoid receptor (CB1), by regulating the ubiquitination and proteasomal degradation of Rap1GAP, activates Rap1 to induce neurite outgrowth in Neuro-2A cells [19], although the exact mechanisms of CB1-induced Rap1GAP ubiquitination and degradation remain unknown. In addition to Rap1GAP, another member of the RapGAP family, SPAR, is also regulated by ubiquitin-proteasome pathway. β-TrCP can target SPAR for degradation in neurons. However, SPAR degradation by β-TrCP depended on the activity-inducible protein kinase Polo-like kinase 2 (Plk2) [20].

Rap1GAP protein can be regulated at multiple post-translational levels. Protein kinase A (PKA) phosphorylates Rap1GAP at Ser441 and Ser499 in response to activation of D1 dopamine receptors, which leads to its inhibition and subsequent activation of Rap1 [10]. Phosphorylation of Rap1GAP is likely to play an important role in regulating medium spiny neuron function through the ability of the dopamine/cAMP/PKA signaling pathway to regulate Rap1 activity [10]. Other kinases, such as CDK1, have also been reported to phosphorylate Rap1GAP at Ser484 during mitosis, but this phosphorylation does not affect the Rap1GAP stimulation of the GTPase activity of Rap1 by Rap1GAP [18]. Our results showed Ser484 phosphorylation was not essential for Rap1GAP degradation in mitosis. One possibility is that Ser484 phosphorylation might play a role in regulating the interaction of Rap1GAP with other proteins, but elucidation of the significance of CDK1-mediated Rap1GAP phosphorylation must await further studies. Furthermore, in unpublished work we have found that Rap1GAP phosphorylation might be reversed by dephosphorylation. The serine/threonine phosphatases, PPM1A and PPM1B, were two of the most abundant proteins co-purified from Rap1GAP complex by tandem affinity purification (TAP)-mass spectrometry. It is known that β-TrCP-dependent degradation of the transcriptional factor Snail was triggered by GSK3β-mediated phosphorylation, and the small C-terminal domain phosphatase (SCP) is a specific phosphatase for Snail. By antagonizing GSK3β-mediated phosphorylation, SCP can stabilize Snail [21]. It will be interesting to test whether PPM1A or PPM1B-mediated Rap1GAP dephosphorylation antagonizes PLK1-mediated Rap1GAP phosphorylation. Future studies are needed to further explore the complex regulation of Rap1GAP proteins and whether dysregulation of Rap1GAP plays a role in tumorigenesis.

## References

[1] Raaijmakers JH, Bos JL (2009) Specificity in Ras and Rap signaling. J Biol Chem 284: 10995–10999. 19091745 10.1074/jbc.R800061200 PMC2670103

[2] Bos JL, de Rooij J, Reedquist KA (2001) Rap1 signalling: adhering to new models. Nat Rev Mol Cell Biol 2: 369–377. 11331911 10.1038/35073073

[3] Zhang Z, Mitra RS, Henson BS, Datta NS, McCauley LK, et al (2006) Rap1GAP inhibits tumor growth in oropharyngeal squamous cell carcinoma. Am J Pathol 168: 585–596. 16436672 10.2353/ajpath.2006.050132 PMC1606505

[4] Kim WJ, Gersey Z, Daaka Y (2012) Rap1GAP regulates renal cell carcinoma invasion. Cancer Lett 320: 65–71. 22266190 10.1016/j.canlet.2012.01.022 PMC3319804

[5] Zheng H, Gao L, Feng Y, Yuan L, Zhao H, et al (2009) Down-regulation of Rap1GAP via promoter hypermethylation promotes melanoma cell proliferation, survival, and migration. Cancer Res 69: 449–457. 19147557 10.1158/0008-5472.CAN-08-2399

[6] Zhang L, Chenwei L, Mahmood R, van Golen K, Greenson J, et al (2006) Identification of a putative tumor suppressor gene Rap1GAP in pancreatic cancer. Cancer Res 66: 898–906. 16424023 10.1158/0008-5472.CAN-05-3025

[7] Tsygankova OM, Prendergast GV, Puttaswamy K, Wang Y, Feldman MD, et al (2007) Downregulation of Rap1GAP contributes to Ras transformation. Mol Cell Biol 27: 6647–6658. 17646383 10.1128/MCB.00155-07 PMC2099240

[8] Zuo H, Gandhi M, Edreira MM, Hochbaum D, Nimgaonkar VL, et al (2010) Downregulation of Rap1GAP through epigenetic silencing and loss of heterozygosity promotes invasion and progression of thyroid tumors. Cancer Res 70: 1389–1397. 20124489 10.1158/0008-5472.CAN-09-2812 PMC2822891

[9] Banerjee R, Mani RS, Russo N, Scanlon CS, Tsodikov A, et al (2011) The tumor suppressor gene Rap1GAP is silenced by miR-101-mediated EZH2 overexpression in invasive squamous cell carcinoma. Oncogene 30: 4339–4349. 21532618 10.1038/onc.2011.141 PMC3154567

[10] McAvoy T, Zhou MM, Greengard P, Nairn AC (2009) Phosphorylation of Rap1GAP, a striatally enriched protein, by protein kinase A controls Rap1 activity and dendritic spine morphology. Proc Natl Acad Sci U S A 106: 3531–3536. 19218462 10.1073/pnas.0813263106 PMC2651273

[11] Tsygankova OM, Feshchenko E, Klein PS, Meinkoth JL (2004) Thyroid-stimulating hormone/cAMP and glycogen synthase kinase 3beta elicit opposing effects on Rap1GAP stability. J Biol Chem 279: 5501–5507. 14660640 10.1074/jbc.M305824200

[12] Polakis P, Rubinfeld BM, McCormick F (1992) Phosphorylation of Rap1GAP in vivo and by cAMP-dependent kinase and the cell cycle p34cdc2 kinase in vitro. J Biol Chem 267: 10780–10785. 1587853

[13] Hershko A, Ciechanover A (1998) The ubiquitin system. Annu Rev Biochem 67: 425–479. 9759494 10.1146/annurev.biochem.67.1.425

[14] Vodermaier HC (2004) APC/C and SCF: controlling each other and the cell cycle. Curr Biol 14: R787–R796. 15380093 10.1016/j.cub.2004.09.020

[15] Skaar JR, Pagan JK, Pagano M (2013) Mechanisms and function of substrate recruitment by F-box proteins. Nat Rev Mol Cell Biol 14: 369–381. 23657496 10.1038/nrm3582 PMC3827686

[16] Frescas D, Pagano M (2008) Deregulated proteolysis by the F-box proteins SKP2 and beta-TrCP: tipping the scales of cancer Nat Rev Cancer. 8: 438–449. 10.1038/nrc2396 PMC2711846 18500245

[17] Elia AE, Cantley LC, Yaffe MB (2003) Proteomic screen finds pSer/pThr-binding domain localizing PLK1 to mitotic substrates. Science 299: 1228–1231. 12595692 10.1126/science.1079079

[18] Janoueix-Lerosey I, Fontenay M, Tobelem G, Tavitian A, Polakis P, et al (1994) Phosphorylation of Rap1GAP during the cell cycle. Biochem Biophys Res Commun 202: 967–975. 8048970 10.1006/bbrc.1994.2024

[19] Jordan JD, He JC, Eungdamrong NJ, Gomes I, Ali W, et al (2005) Cannabinoid receptor-induced neurite outgrowth is mediated by Rap1 activation through G(alpha)o/i-triggered proteasomal degradation of Rap1GAPII. J Biol Chem 280: 11413–21. 15657046 10.1074/jbc.M411521200

[20] Ang XL, Seeburg DP, Sheng M, Harper JW (2008) Regulation of postsynaptic RapGAP SPAR by Polo-like kinase 2 and the SCFbeta-TRCP ubiquitin ligase in hippocampal neurons. J Biol Chem 283: 29424–29432. 18723513 10.1074/jbc.M802475200 PMC2570879

[21] Wu Y, Evers BM, Zhou BP (2009) Small C-terminal domain phosphatase enhances snail activity through dephosphorylation. J Biol Chem 284: 640–648. 19004823 10.1074/jbc.M806916200 PMC2610500

